# The way to a man’s heart is through his stomach: a case of myocardial infarction mimic and pseudo-tamponade in a polytrauma patient

**DOI:** 10.1186/s13049-021-00911-4

**Published:** 2021-07-31

**Authors:** Mathew Brun, Shane Brun, David Pearson, Martin Wullschleger

**Affiliations:** 1 Adult Intensive Care, Unit Gold Coast University Hospital, 1 Hospital Blvd, Southport, Queensland 4215 Australia; 2 Medical Education Unit Gold Coast University Hospital, 1 Hospital Blvd, Southport, Queensland 4215 Australia; 3 Trauma Service Royal Brisbane and Women’s Hospital, Herston, Queensland 4029 Australia

**Keywords:** Trauma, Polytrauma, Multi-trauma, Anticoagulation, Thrombolysis, Haematoma, Pseudotamponade, Tamponade, STEMI, Mimic

## Abstract

**Background:**

There exists a therapeutic conflict between haemorrhage control and prevention of thromboembolic events following polytrauma and complications are not uncommon. Such opposing therapies can result in unexpected pathophysiology and there is a real risk of misdiagnosis resulting in harm. This case presents a previously unreported complication of prevention and management of thromboembolism- STEMI (ST elevation myocardial infarction) and tamponade mimic secondary to retroperitoneal haematoma.

**Case presentation:**

We present a 50-year-old male polytrauma patient who following treatment for presumed pulmonary embolus demonstrated classical clinical findings of myocardial infarction and pericardial tamponade secondary to a retroperitoneal haematoma. This is an event not previously reported in the literature. The risk of adverse outcome by management along the standard lines of STEMI (ST elevation myocardial infarction) was averted through awareness for alternative aetiology via a multi-team approach which resulted in percutaneous drainage of the haematoma and complete resolution of symptoms.

**Conclusions:**

This manuscript highlights the therapeutic conflict between haemorrhage control and prevention of thromboembolic events in critically injured, the importance of high index of suspicion in this patient cohort and the benefits of multidisciplinary decision making in the complex patient through a not previously published pathophysiologic phenomenon.

## Introduction

Trauma patients are at high risk for venous thrombosis the consequences of which can be catastrophic for survivors [1]. In the multiply injured patient, the clinician must balance the risks of both haemorrhage and thrombosis when commencing prophylactic anticoagulation. In the event of a major thrombotic complication, such as a pulmonary embolus (PE), therapeutic anticoagulation and even thrombolysis [2, 3] must then be considered further heightening the risk of haemorrhagic complications. The haemorrhagic complications of anticoagulation and thrombolysis include, but are not limited to, anaemia requiring transfusion, intracranial haemorrhage, haemothorax, haemorrhagic pericardial effusion, death from haemorrhage and retroperitoneal haematoma [4]. Whilst rare, the incidence of retroperitoneal haematoma is increasing because of more patients taking antiplatelet and anticoagulant medications [5]. Complications of retroperitoneal haematoma (outside haemorrhage) are rare and largely undocumented [1, 5] the manifestations vary, requiring a high index of suspicion. Abdominal compartment syndrome and femoral neuropathy [5, 6] are more commonly cited, with less common examples of ileus [7], bowel obstruction [8], obstructive uropathy [9] and an appendicitis mimic [10] in the literature.

**Fig. 1:**
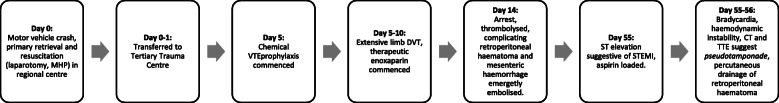
Case timeline

The case presented outlines the sequence of events following a motor vehicle crash (summarised in Fig. 1). A man sustained multiple injuries and was transferred from a regional hospital to a tertiary trauma centre for definitive care. Fourteen days into his stay the patient was thrombolysed during an in-hospital cardiac arrest for presumed pulmonary embolus. Thrombolysis was complicated by a large retroperitoneal haematoma with mass effect, resulting in gastric obstruction, manifesting as an inferior myocardial infarction mimic through cardiac compression.

## Case description

A 50-year-old Caucasian male, was retrieved from a regional hospital to our tertiary trauma centre following a high velocity motor vehicle crash. Prior to his retrieval he had undergone damage control surgery, including a resuscitative laparotomy, and fixation of limb fractures. He had received a massive haemorrhage protocol (MHP) as part of a red blanket protocol. His presenting injuries at the time included: small volume intracranial haemorrhage and major limb fractures (the cause for his refractory shock in retrospect). There was no spinal injury and no major intrathoracic, intra-abdominal or pelvic injuries. His injury severity score (ISS) was 35. The extent of his lower limb injuries, large volume MHP including tranexamic acid and immobility placed him at high risk of venous thrombosis. His thrombotic risk was balanced against the presence of intracranial haemorrhage and under guidance of Neurosurgery prophylactic heparin was not commenced until after an unchanged day five CT of the head. His Intensive Care Unit (ICU) admission was complicated by delirium and extensive deep vein thrombus (clot burden in both upper and lower limbs). He was discharged from the ICU on day 14 having been commenced on therapeutic enoxaparin, he sustained a PEA cardiac arrest the same day. In the setting of acute deterioration and known extensive clot burden he was presumptively thrombolysed for PE.

A Computed Tomography Pulmonary angiogram (CTPA) and contrast Computed Tomography (CT) abdomen shortly after his cardiac arrest and thrombolysis revealed mesenteric haemorrhage and a large retroperitoneal haematoma in the pararenal space, likely a complication of the thrombolysis. There were no PE identified on the study. His superior mesenteric artery was emergently embolised and the retroperitoneal haematoma managed conservatively. Following the resuscitative phase of his readmission his clinical status improved now some 30 days following his initial presentation. This was punctuated by a slow ventilator wean, unexplained sinus tachycardia, critical illness myopathy, poor oral intake and an agitated delirium. On follow up contrast CT of the abdomen the haematoma measured 22 × 8 × 23 (TV x AP x CC) cm and had no interval change on subsequent imaging.

**Fig. 2:**
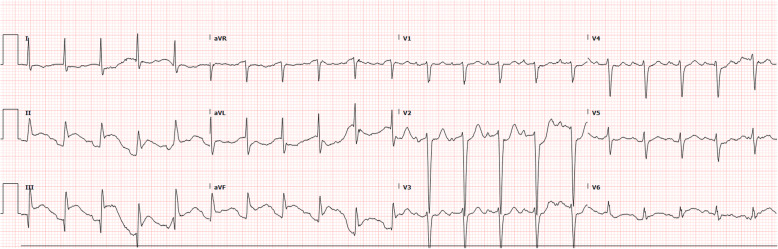
ECG showing STE > 1 mm in leads II, III and aVF with STE III > II and subtle STD in I suggestive of RCA occlusion

Poor oral intake had been problematic for this patient. With the importance of protein energy intake in a hypermetabolic post-trauma state nasogastric tube (NGT) was placed to supplement calories. The NGT fell victim to the patient’s delirium on multiple occasions and replacement proved difficult, requiring endoscopic placement and periods where he was NGT free. Twenty-five days into his second ICU stay and 55 days post initial trauma, he was noted to have a large gastric bubble and an NGT was replaced and again promptly removed by the patient. In the early morning of the 26th day he was noted to have ST elevation suggestive of inferior myocardial infarction, most suggestive of right coronary artery territory (with both inferior and right ventricular pattern of change)- (Fig. 2).

**Fig. 3:**
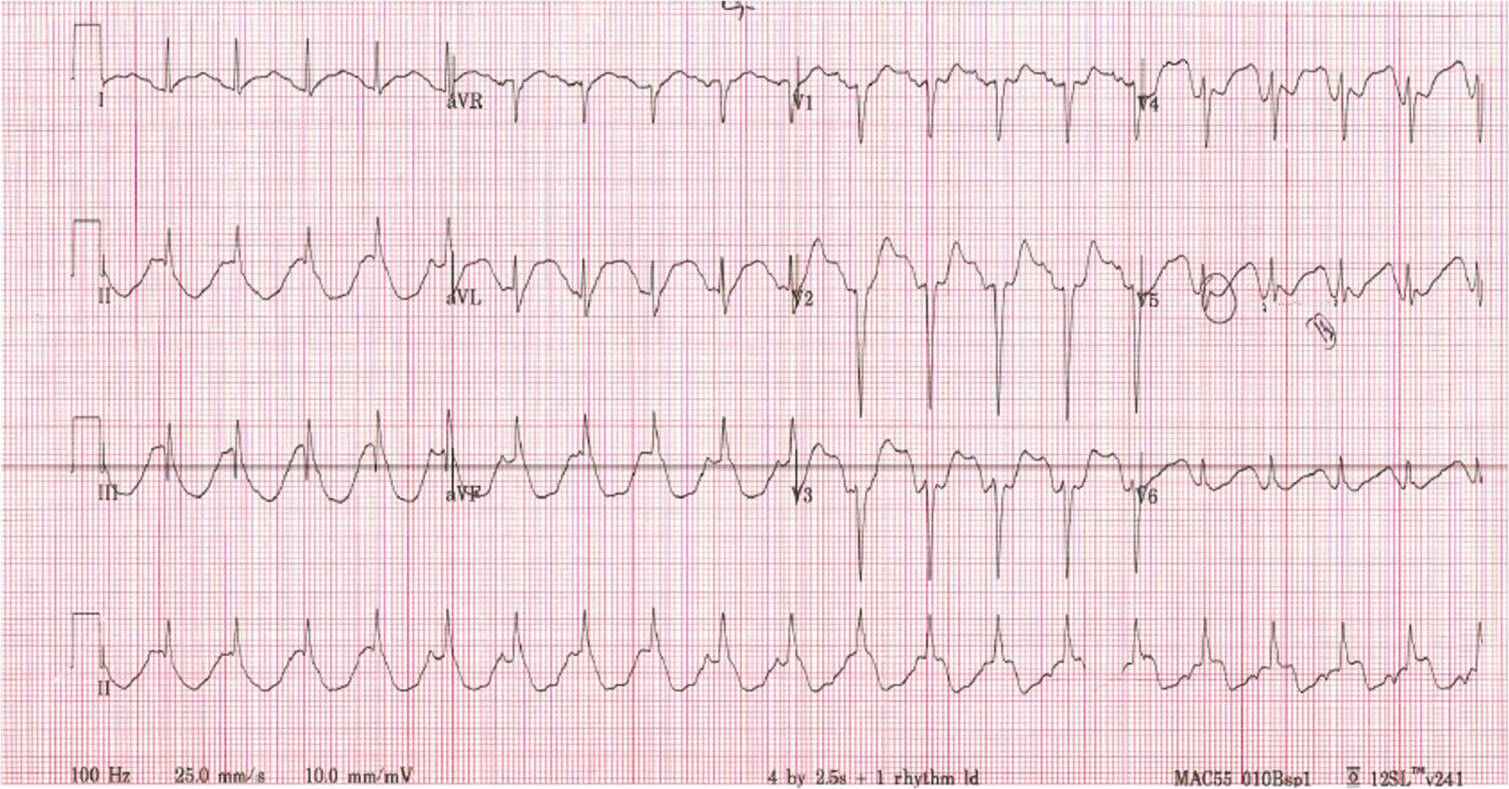
Bizarre narrow complex tachycardia with large U waves in lateral leads and prolonged QT

**Fig. 4:**
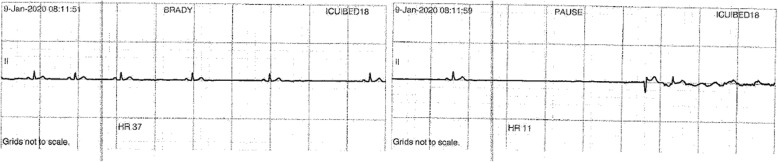
Telemetry showing progress from sinus bradycardia, to severe bradycardia then sinus pause

Delirium made assessment for chest pain difficult with the patient becoming increasingly agitated. Aside from his sinus tachycardia, his blood pressure and mean arterial pressure (MAP) were initially within normal range and he had no clinical evidence of heart failure. A loading dose of 300 mg of aspirin was administered whilst awaiting serial high sensitivity troponin (hsTNI), which were subsequently negative. Full blood count and electrolytes were all within normal range. His Electrocardiogram (ECG) evolved much like inferior myocardial infarction pattern (complicated by right ventricular involvement) over the next 2 h as shown in Figs. 3 and 4.

During this time a worsening bradycardia evolved with progressive haemodynamic instability. The patient became less alert, with episodes of profound hypotension (MAP as low as 30 mmHg). An adrenaline infusion was rapidly up-titrated as differentials were considered.

An urgent CT with contrast of the abdomen was performed along with transthoracic echo (TTE) undertaken by the cardiology service.

**Fig. 5:**
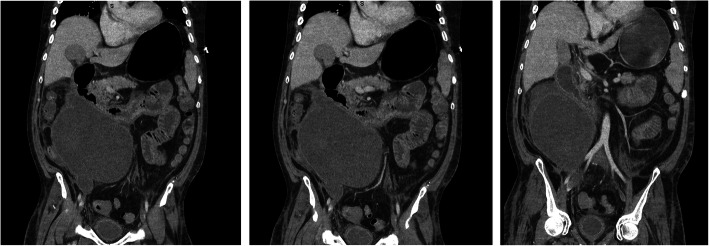
Coronal CT sections showing a large retroperitoneal haematoma, duodenal compression and gastric distension and a distorted left ventricle

Contrast CT revealed interval increase in the size of the haematoma, compression of D3 (distal duodenum) and resultant gastric distension causing left ventricular distortion (Fig. 5).

**Fig. 6:**
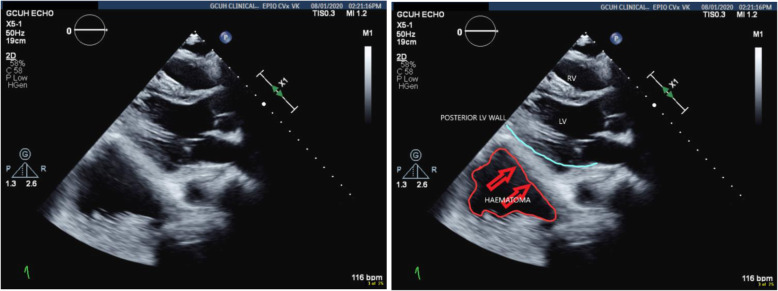
Parasternal long axis view showing the haematoma compressing the posterior wall of the LV

**Fig. 7:**
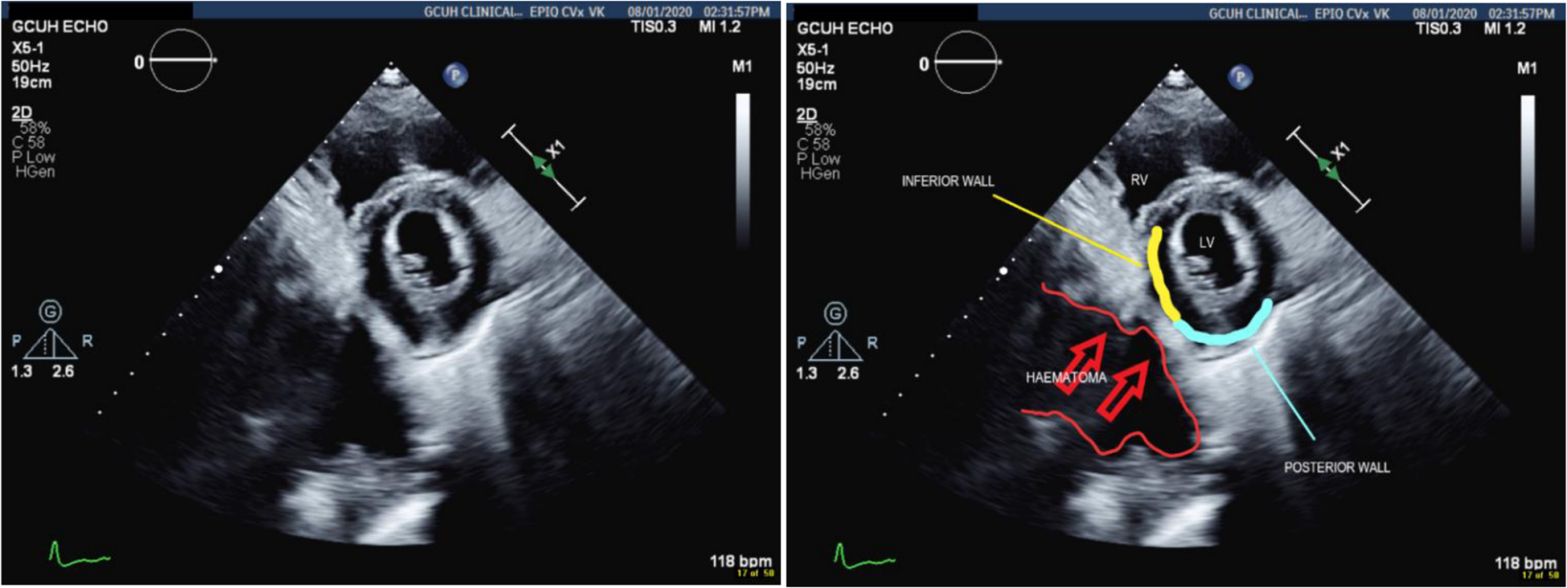
Short axis view showing the haematoma compressing both posterior and inferior walls of the LV

TTE confirmed retrocardiac compression as a result of the expanding retroperitoneal haematoma (Figs. 6 and 7).

The above findings were presented in a group setting to staff specialists from Intensive Care, Trauma, Cardiology, Interventional Radiology and Cardiac Anaesthetics. An explanation for the patient’s deterioration thought most likely to be a result of:

…*the combined mass effect of the haematoma and gastric bubble resulting in a STEMI mimic. Additionally, there was haemodynamic compromise through extra-cardiac compression- “pseudo-tamponade”, and impaired venous return from vena caval compression by the haematoma…*

The haematoma was rapidly drained by the Interventional Radiologist yielding more than 1500 mL of liquefied haematoma at insertion. This resulted in a return to normal sinus rhythm, no further ECG changes and a swift resolution of shock. A later coronary angiogram showed no stenotic lesions removing the possibility of coronary artery disease contributing to the pathophysiology.

## Discussion

Retroperitoneal haematoma presents in trauma as primary pathology and is also well recognised as a complication of anticoagulation, thrombolysis and interventional procedures [11]. Clinicians must have a high index of suspicion, as there may only be subtle signs such as anaemia and tachycardia (both very non-specific in the trauma patient). Manifestations of mass effect already described are rare and varied, and, as can be seen in this case, may mimic other pathology.

This case is an unusual complication of a retroperitoneal haematoma and highlights some of the complexities of anticoagulation and thrombolysis in the trauma patient. Additionally, its manifestation as an inferior STEMI mimic and extracardiac compression (*pseudo-tamponade*) may well be the first recognised in the literature.

The expanding haematoma and associated inflammatory change resulted in compression of the duodenum and subsequent gastric distension. Failure to decompress the stomach may have contributed to cardiac compression. When combined with an expanding haematoma simultaneously applying direct force on the heart and compressing the inferior vena cava, we were faced with a patient showing signs of myocardial infarction and cardiac tamponade.

STEMI can present in the trauma patient and medical management carries similar haemorrhage risk to the management of VTE (venous thromboembolus). There are some classic ECG mimics of ST elevation that should be considered (Table 1). Specific regional patterns, like the inferior pattern seen in our patient, are rare (Table 2).

**Table 1:** Classic ST elevation mimics

Hyperkalaemia	
Early repolarisation	
Brugada pattern	
Left ventricular hypertrophy	
Left bundle branch block	
Ventricular aneurysm	
Myopericarditis	
Hypothermia- J or Osborne wave	
Vasospasm	
Elevated intracranial pressure	
Takutsubo cardiomyopathy

**Table 2:** Rare and case reported ST elevation mimics

Pneumopericardium [12]	
Regional pericarditis [13]	
Severe hypocalcaemia [14]	
Acute pancreatitis [15]	
Pulmonary embolus [16]	
Intermittent delta wave [17]	
Type-A aortic dissection [18]	

In this patient’s case, he was administered aspirin prior to hsTNI being available, it is not uncommon to initiate treatment with typical ECG findings of STEMI. The administration of aspirin is likely to have further increased haemorrhage risk.

Conservative management of retroperitoneal haematomas is recognised however there is still a lack of level I evidence [1, 19]. Consequently, clinicians must remain vigilant for potential haematoma expansion. This risk increases significantly in the long stay trauma patient who is likely to be on prophylactic anticoagulation.

As the haemorrhage is contained, the changes may be subtle and manifestations of mass effect may be a late sign mimicking other pathology. In the absence of significant anaemia or mass effect the threshold for drainage (if appropriate at all) remains unclear. The question of surveillance is also unanswered. Contrast CT is the preferred modality of investigation but carries with it the risk of ionising radiation and contrast burden [1]. Additionally, there are the risks associated with intrahospital transport in most settings.

Given the unique presentation a multidisciplinary decision-making process was indicated. Input from cardiology, trauma services, cardiac anaesthetics and interventional radiology allowed complex pathophysiology to be identified and safe planning of intervention.

The multi-trauma patient is complex with competing pathology and therapeutic modalities. Whilst their presentations are more often purely surgical, they are at risk of medical complications during admission.

This case demonstrates an interesting interplay between a complication of treatment for medical pathology (thrombolysis for PE) evolving into an unusual surgical consequence which masqueraded as a medical event. Such complex pathophysiology demands a multidisciplinary approach and more than one investigative modality.

## Conclusion

This case highlights some of the risks associated with the management of vein thrombosis and VTE in the polytrauma patient. Whilst rare, retroperitoneal haematomas are being recognised more frequently. The apparent increase may likely be a direct consequence of more interventional procedures being performed and an increasing number of patients on antiplatelet and anticoagulant medications. A high index of suspicion is important when managing the polytrauma patient and consideration given to surveillance in the setting of a known haematoma. The incidence of varied and novel pathophysiologic effects is somewhat unknown and should be anticipated when interpreting clinical findings and in considering specialty consultation, investigation and management.

## Data Availability

Not applicable.

## Abbreviations

STEMI: ST elevation myocardial infarction
PE: Pulmonary embolus
MHP: Massive haemorrhage protocol
DVT: Deep vein thrombosis
ISS: Injury severity score
ICU: Intensive Care Unit
CTPA: Computed Tomography Pulmonary angiogram
CT: Computed Tomography
NGT: Nasogastric tube
hsTNI: High sensitivity troponin
ECG: Electrocardiogram
MAP: Mean arterial pressure
TTE: Transthoracic echo
VTE: Venous thromboembolus

## References

[1] Hölting T, Buhr HJ, Richter GM, Roeren T, Friedl W, Herfarth C (1992). Diagnosis and treatment of retroperitoneal hematoma in multiple trauma patients. Arch Orthop Trauma Surg.

[2] Nathens AB, McMurray MK, Cuschieri J (2007). The practice of venous thromboembolism prophylaxis in the major trauma patient. J Trauma.

[3] Golob JF, Sando MJ, Kan JC, Yowler CJ, Malangoni MA, Claridge JA (2008). Therapeutic anticoagulation in the trauma patient: is it safe?. Surgery..

[4] Brathwaite CEM, Mure AJ, O’Malley KF, Spence RK, Ross SE (1993). Complications of anticoagulation for pulmonary embolism in low risk trauma patients. Chest..

[5] González C, Penado S, Llata L, Valero C, Riancho JA (2003). The clinical Spectrum of retroperitoneal hematoma in anticoagulated patients. Medicine..

[6] Parmer SS, Carpenter JP, Fairman RM, Velazquez OC, Mitchell ME (2006). Femoral neuropathy following retroperitoneal Haemorrhage: case series and review of the literature. Ann Vasc Surg.

[7] Drew JH (1973). Ileus in retroperitoneal hæmatoma. Med J Aust.

[8] Chatzis I, Katsourakis A, Noussios G, Chouridis P, Chatzitheoklitos E. Delayed Small Bowel Obstruction after Blunt Abdominal Trauma. A Case Report. 2016;108(5):597–9. 10.1080/00015458.2008.11680295. 10.1080/00015458.2008.11680295 19051476

[9] Kluger Y, Altman GT, Deshmukh R, Townsend RN, Diamond DL (1993). Acute obstructive uropathy secondary to pelvic hematoma compressing the bladder: report of two cases. J Trauma.

[10] Jurisic D, Doko M, Glavan E, Vidovic D, Matkovic K, Pitlovic V (2006). Spontaneous retroperitoneal haematoma associated with clopidogrel therapy mimicking acute appendicitis. Br J Clin Pharmacol.

[11] Feliciano DV (1990). Management of traumatic retroperitoneal hematoma. Ann Surg.

[12] Ratnayake EC, Premaratne S, Lokunarangoda N, Fernando S, Fernando N, Ponnamperuma C, Santharaj WS (2015). Pneumopyopericardium mimicking an inferior ST elevation myocardial infarction with regional electrocardiogram changes: a case report. BMC Res Notes.

[13] Youssef G, Khouzam S, Sprung J, Bourke DL (2001). Regional pericarditis mimicking myocardial infarction. Anesthesiology..

[14] Adeel MY, Clarke J-RD, Shetty S, Arora A, Buscher MG. Severe hypocalcemia mimicking acute inferior ST-segment elevation myocardial infarction. Oxford Med Case Rep. 2018;2018(12). 10.1093/omcr/omy103. 10.1093/omcr/omy103 PMC6247139 30487989

[15] Yu ES, Lange JJ, Broor A, Kutty K (2019). Acute pancreatitis masquerading as Inferior Wall myocardial infarction: a review. Case Rep Gastroenterol.

[16] Zhan Z-Q, Wang C-Q, Wang Z-X (2015). Diagnosing acute pulmonary embolism masquerading as inferior myocardial infarction. Am J Emerg Med.

[17] Bolognesi M. Case Report Cardiovascular Disorders and Medicine Intermittent delta waves mimics inferior myocardial infarction in young athlete. 2016;2(1):1–2. 10.15761/CDM.1000119.

[18] Wang W, Wu J, Zhao X, You B, Li C (2019). Type-a aortic dissection manifesting as acute inferior myocardial infarction. Medicine..

[19] Chan YC, Morales JP, Reidy JF, Taylor PR (2008). Management of spontaneous and iatrogenic retroperitoneal haemorrhage: conservative management, endovascular intervention or open surgery?. Int J Clin Pract.

